# Effects of sociodemographic and health factors on the self-management of non-communicable diseases among Chilean adults during the Covid-19 pandemic

**DOI:** 10.1371/journal.pgph.0000763

**Published:** 2022-07-21

**Authors:** Daniela Nicoletti-Rojas, Rodrigo Retamal, Ricardo Cerda-Rioseco, Lorena Rodríguez-Osiac, Mauricio Fuentes-Alburquenque, Marcela Araya-Bannout

**Affiliations:** 1 Departamento de Nutrición, Facultad de Medicina, Universidad de Chile, Santiago, Región Metropolitana, Chile; 2 Programa de Doctorado en Salud Pública, Escuela de Salud Pública, Facultad de Medicina, Universidad de Chile, Santiago, Región Metropolitana, Chile; 3 Departamento de Antropología, Facultad de Ciencias Sociales, Universidad de Chile, Santiago, Región Metropolitana, Chile; 4 Departamento de Ciencias de la Salud, Facultad de Medicina, Pontificia Universidad de Chile, Santiago, Región Metropolitana, Chile; 5 Escuela de Salud Pública, Facultad de Medicina, Universidad de Chile, Santiago, Región Metropolitana, Chile; 6 Departamento de Promoción de la Salud de la Mujer y el Recién Nacido, Facultad de Medicina, Universidad de Chile, Santiago, Región Metropolitana, Chile; South African Medical Research Council, SOUTH AFRICA

## Abstract

Individuals with non-communicable diseases (NCDs) are potentially at increased vulnerability during the Covid-19 pandemic and require additional help to reduce risk. Self-management is one effective strategy and this study investigated the effect of sociodemographic and health factors on the self-management of some non-communicable diseases, namely hypertension, type 2 diabetes mellitus and dyslipidemia, among Chilean adults during the Covid-19 pandemic. A cross-sectional telephone survey was carried out on 910 participants with NCDs, from Santiago, Chile. An adapted and validated version of the “Partners in Health” scale was used to measure self-management. Exploratory Factor analysis yielded five dimensions of this scale: Disease Knowledge, Healthcare Team Relationship, General Self-Management and Daily Routines, Drug Access and Intake, and Monitoring and Decision-Making. The average of these dimensions was calculated to create a new variable Self-Management Mean, which was used as a dependent variable together with the five separate dimensions. Independent variables included age, gender, years of schooling, number of diseases, the percentage of Multidimensional Poverty Index in the commune of residence, and self-rated health status. Beta regressions and ANOVA for the Beta regression residuals were utilized for analyses. Beta regression model explained 8.1% of the variance in Self-Management Mean. Age, years of schooling, number of diseases and self-rated health status were statistically associated with Self-Management Mean and dimensions related to daily routines and health decision making, such as Disease Knowledge, General Self-Management and Daily Routines, and Monitoring and Decision-Making. Gender and the percentage of Multidimensional Poverty Index in the commune of residence were insignificant. Strategies for self-management of NCDs during a crisis should consider age, years of schooling, number of diseases, and self-rated health status in their design.

## Introduction

Currently global health is characterized by a high prevalence of non-communicable diseases [1] and the emergence of new infectious diseases such as Covid-19 with the resulting global pandemic [2]. Preventing and controlling infectious diseases, nutritional problems and associated chronic diseases require pertinent planning and strategies [3]. Individuals living with non-communicable diseases (NCDs) [4] are particularly vulnerable as a result of the pandemic. Covid-19 mortality rates reported worldwide have shown higher rates of morbidity, hospitalization and mortality in particularly for Type 2 diabetes and arterial hypertension, as well as cancer [5–7]. In addition, measures used to reduce the spread of Covid-19 including social distancing, mobility restrictions and lockdowns may impact on people with NCDs by making it more difficult to access health services and to follow prescribed diets or perform physical activity, as well as increasing smoking and excessive alcohol consumption [8]. In Chile, the 10^th^ Report of Covid-19 Symptoms and Practices National Monitoring (in Spanish, Monitoreo Nacional de Síntomas y Prácticas Covid-19, or MOVID-19) [9], showed that only 26.1% those with NCDs had access to healthcare services due to the lockdown and the reduction in routine health services. The lack of programmatic alignments for managing the NCD population during the Covid-19 pandemic may obstruct the fight against both pandemics, especially in low and middle income countries, and therefore, the challenge to keep healthcare continuity for chronic disease is relevant [8].

In Chile the primary healthcare system aims at promoting healthy lifestyle habits and capacities for individuals and communities. All risk factors related to lifestyle habits, such as diet, physical activity, and smoking, among others, constitute an important part of the burden of disease in Chile. The challenge of the primary healthcare system is to achieve changes in the behavior of individuals to improve lifestyle habits, to develop protecting factors, and to decrease the prevalence of risk factors. The lockdown has impacted on various primary healthcare programs [10], such as the Cardiovascular Health Program (in Spanish, Programa de Salud Cardiovascular, PSCV onwards), which aims to prevent and reduce morbidity, disability and premature mortality caused by cardiovascular diseases (arterial hypertension, type 2 diabetes mellitus and dyslipidemia) [11]. Implementation of strategies to transfer knowledge and skills to those with NCDs so that they can manage their diseases, improve health and quality of life, and prevent saturation of the healthcare system is important [12, 13].

One strategy that has shown some effectiveness in controlling NCDs is self-management [14] which is defined as a set of knowledge, attitudes, skills and behaviors that individuals develop to cope with their experience including identification and management of symptoms, treatment adherence, prevention of physical, social and emotional consequences, as well as adapting to these consequences [15–17]. Self-management is developed with the support of healthcare professionals, the healthcare system and other sectors [18, 19].

Self-management is a complex phenomenon determined by multiple factors (medical, biological, sociodemographic, and psychological, among others) [20–22], which may undermine or enhance self-management. To develop and promote self-management, it is necessary to know what factors influence self-management in the NCD population in the context of the Covid-19 pandemic, as well as a reformulation of the tasks and roles of healthcare teams. The Chilean primary healthcare system is a well-developed public system to support healthcare in an individual and community level [23]. It is possible that the knowledge of the effect of selected sociodemographic and health factors on self-management may help to Chilean and other countries to adjust and re-design programs aimed to provide support for self-management to the NCDs population during sanitary crisis such as the Covid-19 pandemic. Additionally, it may help to other countries that have primary healthcare systems to develop more resilient programs to the NCDs population during sanitary crisis. The objective of the present study is to investigate the effect of selected sociodemographic and health factors on NCD self-management.

## Subjects and methods

### Study design

**Fig 1:**
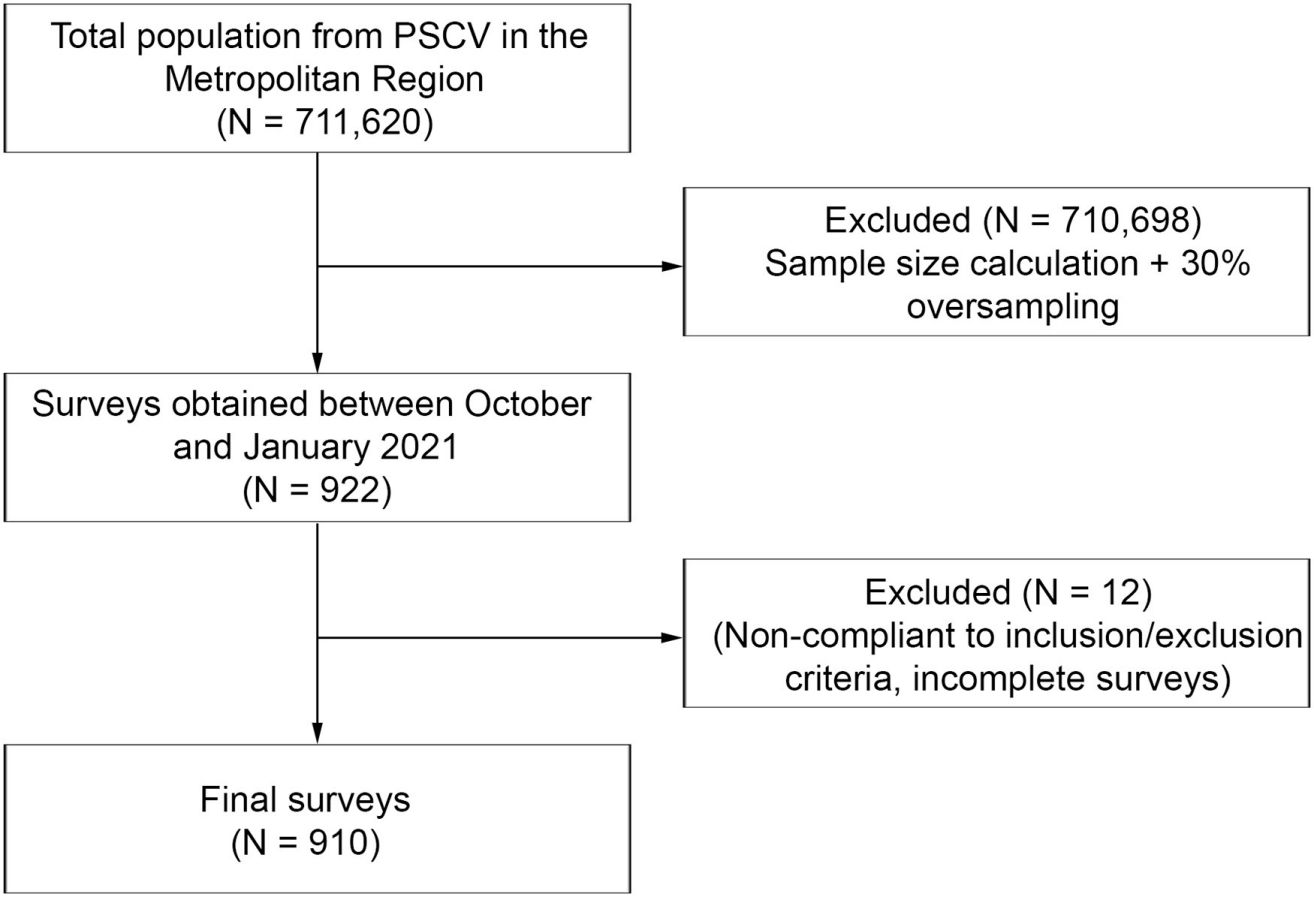
Sample flow chart.

The sample size calculation for proportions indicated a minimum sample size of 666 participants from the users of the PSCV in the Metropolitan Region (N = 711,620), based on alpha of 5%, beta of 80% and 99% confidence interval (Fig 1) and oversampling by 30% to yield a minimum sample of 900 individuals. Due to the Covid-19 lockdown restrictions data collection was performed via a telephone survey.

Participants in the study were selected using a three-staged probabilistic design. In the first stage, the 32 communes that comprise the city of Santiago were divided in three tertiles utilizing the percentage of the Multidimensional Poverty Index (%MPI) of each commune. Three communes were randomly selected from each tertile, giving a total of 9 communes. In the second stage, two Family Health Care Centers (Centro de Salud Familiar or CESFAM) were randomly selected from each of these communes. In the third stage, individuals were selected from each CESFAM through a stratified random sampling by age and gender.

The inclusion criteria were people enrolled in the PSCV, to be older than 20 years of age, with access to a telephone or to be able to connect via video call. Exclusion criteria considered people that had difficulties to answer a phone call or to respond to the survey appropriately, such as participants with hearing, cognitive or mental health disabilities.

### Data collection

Due to the lockdown the survey was carried out by telephone. The decision of carrying out the survey by telephone was based on the fact that this was the unique source of contact to the participants. Surveys carried out by telephone have proven better response rates compared to internet surveys [24, 25]. In order to allow for non-response a total of 7,282 telephone numbers (landline and mobile) were obtained from the selected CESFAM. The survey was carried out between October 2020 and January 2021 (14 weeks). The phone numbers were divided between nine experienced and previously trained telephone operators, who made an average of 719 calls per week, averaging 222 effective calls per week. Informed consent was obtained before the start of the survey. The average call duration was 37 minutes (SD = 14 minutes) with a minimum duration of 10 minutes and a maximum duration of 112 minutes. Each call was recorded. Ineffective calls corresponded to either wrong or invalid numbers. The total number of effective calls gave a weekly average of 66 responses, the rest corresponded to participants that fell under exclusion criteria or declined to participate. Participants who refused to participate were not different in terms of gender, age, and commune of residence.

The survey obtained data from 922 individuals (477 women and 433 men). A total of twelve participants were excluded (Fig 1) due to non-compliance with the inclusion and exclusion criteria, or for having incomplete responses. Therefore, the final analysis was performed on a total sample of 910 participants.

### Variables

The study utilized the Self-management mean (SMM) and its dimensions as outcomes, while sociodemographic and health self-perception variables were used as independent variables. SMM was built from the “Chronic disease self-management in Covid-19 pandemic scale for people enrolled in the Cardiovascular Health Program”, which was designed based on the “Partners in Health (PIH)” scale developed in 2003 at the Flinders Human Behavior & Health Research Unit at the University of Flinders [26]. The original instrument is an 11-item scale comprising three dimensions: core self-management, condition knowledge and symptom monitoring, and it was designed to be self-administered. All the questions were adapted for telephone usage and placed in the context of the pandemic and the PSCV. The original score for each question was from 0 to 8 and it was modified from 0 to 100% in 10% intervals. The score was inverted, where 0 indicates absence of self-management and 100 indicates maximum possible presence of self-management. These modifications were made following the recommendations of Bandura [27] to measure self-efficacy, taking into consideration that self-efficacy and self-management are strongly related [28] and that in many cases the scales of self-efficacy are used to evaluate self-management [29].

New questions were included aiming to obtain information concerning support networks, including family, community and health teams making a 17 item scale. Finally, the scale contained 17 items (S1 Text).

The scale was submitted to a process of content validation. Six cognitive interviews [30] were performed, with the objective of assessing comprehension and sociocultural pertinence. The interviews were performed on three men and three women, of ages and diagnosis equivalent to the population to which the survey was applied. All participants showed understanding of the statements and the response scale.

The scale was also submitted to a psychometric properties analysis, utilizing an exploratory factor analysis. A total of two items were removed from the original scale, as they showed low commonality (items 8 and 16, S1 Text), which resulted in the final 15-items instrument (Cronbach’s α = 0.836). The exploratory factor analysis provided five dimensions: Disease Knowledge (DK), that refers to the self-reported knowledge beliefs that participants have regarding their condition in general, including causes, effects and relation to Covid-19; Healthcare Team Relationship (HTR), that considers the participant’s engagement in the decision-making process pertaining to treatment and perceived support from the healthcare team; General Self-Management and Daily Routines (SMDR), which refers to everyday management of actions related to treatment, such as diet, physical activity and emotional management; Drug Access and Intake (DAI), related to the possibility of accessing required medication for treatment and correct intake; and Monitoring and Decision-Making (MDM); related to the capacity of participants to self-assess disease signs and symptoms, and to make decisions based on these assessments. Finally, SMM was created using the mean of the five dimensions.

The sociodemographic factors used as independent variables were gender (man and woman), age (subtracting the date of birth to the date of the interview), the %MPI of the commune of residence, and the self-reported years of schooling categorized into five groups (incomplete primary education [< 8 years], incomplete secondary education [< 12 years], complete secondary education [≥ 12 years], complete technical education or incomplete higher education, and complete higher education). The health factors were the self-rated health status (divided in four categories: poor, fair, good and excellent) and the number of diseases that was calculated by adding the number of self-declared diseases attended by the PSCV: diabetes, arterial hypertension, and dyslipidemia. Other diseases such as neoplasia, blood disorders, endocrine diseases, mental or neurologic diseases, digestive diseases, respiratory diseases (not related to Covid-19), musculoskeletal disorders, genitourinary diseases, hearing disorders, infectious diseases (not related to Covid-19), immune diseases, ophthalmologic diseases and disorders, cardiovascular diseases, and metabolic diseases were also included.

### Data analysis

Beta regressions were used to determine the effect of each independent variable on the variability of the dependent variables. Beta regressions have been shown to give better data adjustment when dependent variables present asymmetrical, outlier values, given their higher flexibility when compared to standard linear regressions [31]. In addition, evidence indicates that beta distribution-based analysis is recommended in continuous answer formats, where responses must be marked on a continuous range, with no semantic references, as is the case of this survey [32–34]. Shapiro-Wilk tests performed on residuals from linear and beta regressions using data from this study rejected normal distribution. However, the visualization of residual values (q-q plots, not presented in this paper) and Akaike Information Criteria values (AIC beta regression model = -736.265; AIC linear regression model = -700.099) showed better adjustments in beta regressions compared to linear regressions. Analysis of variance (ANOVA) was performed using the residual values obtained from beta regressions with the aim of controlling for the effect of the other independent variables. Finally, Bonferroni correction was applied to correct for the number of statistical tests undertaken, since six beta regression models were performed using the same dependent variables (p-value<0.007).

### Ethics statement

The Project was approved by the ethical committee of the Faculty of Medicine, University of Chile (Project: Nº 100–2020). Verbal informed consent was obtained from all participants.

## Results

**Fig 2:**
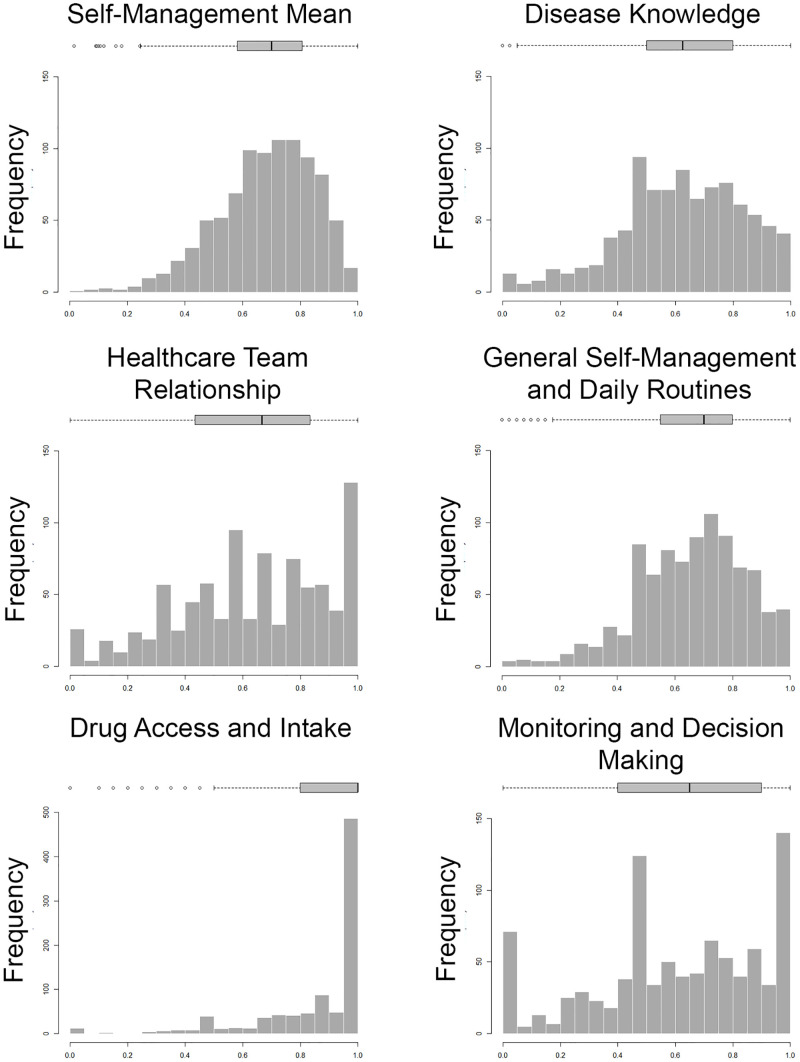
Histogram and box-plot of self-management mean and its dimensions.

Table 1 provides the descriptive statistics and Fig 2 shows histograms and box-and-whiskers charts for the SMM and the dimensions. As can be seen from Table 1 and Fig 2, the general pattern of SMM and the dimensions showed negative skewness, as well as means and medians over 0.6. As can be seen from the interquartile ranges, MDM, HTR and DK dimensions showed high dispersion, while DAI, SMM, and SMDR dimensions showed low dispersion. The DAI dimension was worth highlighting since it presented a median close to the upper limit (>0.999), pronounced asymmetry and low variability in comparison to other dimensions.

**Table 1:** Summary of the dependent variables.

Variable	N	Mean	Median	I.Q.R.	Skewness
Self-Management Mean (SMM)	910	0.682	0.701	0.225	-0.649
Disease Knowledge (DK)	910	0.626	0.625	0.300	-0.494
General Self-Management and Daily Routines (SMDR)	910	0.665	0.700	0.250	-0.619
Healthcare Team Relationship (HTR)	909	0.634	0.666	0.400	-0.449
Drug Access and Intake (DAI)	903	0.873	0.999	0.200	-2.093
Monitoring and Decision-Making (MDM)	910	0.614	0.650	0.499	-0.504

I.Q.R.: interquartile rank.

Table 2 presents descriptive statistics for the independent variables. It was found that women were more likely to participate. The average age was 57.86 years, with a standard deviation of 18.17 years. Minimum and maximum ages were 21 years and 101 years respectively. The majority of participants declared had incomplete secondary education, two diseases, and good health. The %MPI in the commune of residence showed an average of 20.87%, with a standard deviation of 0.06%. The commune with the lowest %MPI was Santiago and the commune with the highest participation was Recoleta.

**Table 2:** Summary of the independent variables.

Variables	Categories	Frequency	Percent
Gender	Female	477	52.42
	Male	433	47.58
Age (years)	20–39	223	24.51
	40–69	420	46.15
	70+	267	29.34
Years of schooling	Primary incomplete	124	13.63
	High school incomplete	221	24.29
	High school complete	249	27.36
	Bachelor incomplete / technical complete	213	23.41
	Bachelor complete	103	11.32
Number of diseases	1	200	21.98
	2	281	30.88
	3	212	23.30
	4	118	12.97
	5 or more	99	10.88
Health status	Poor	72	7.91
	Fair	344	37.80
	Good	412	45.27
	Very good	82	9.01
% Multidimensional Poverty Index by commune	Santiago (9.63%)	117	12.86
	Macul (13.47%)	84	9.23
	La Cisterna (17.82%)	100	10.99
	Recoleta (22.5%)	125	13.74
	Pudahuel (22.51%)	124	13.63
	Puente Alto (23.31%)	107	11.76
	Peñalolén (26.28%)	104	11.43
	Pedro Aguirre Cerda (26.76%)	95	10.44
	Conchalí (29.37%)	54	5.93

Table 3 presents the results of the beta regressions of SMM and the dimensions as dependent variables and their association with the independent variables. Pseudo R-squared showed that the independent variables explained approximately 8.10% of the SMM variation, while the SMDR dimension showed the highest pseudo R-squared value of 13.0%. The variables that were significantly associated with SMM were age, years of schooling, number of diseases, and health status. The DK dimension was significantly associated with gender, years of schooling, number of diseases, and very good health status. The SMDR dimension was significantly associated with age, higher complete education, and health status. The HTR dimension was only significantly associated with very good health status, while the MDM dimension was significantly associated with age and number of diseases. The DAI dimension was not significantly associated with any of the independent variables.

**Table 3:** Beta regressions of self-management mean and its dimensions with the independent variables.

Variable	Self-Management Mean				Disease Knowledge				Healthcare Team Relationship			
	Estimate	S.E.	Z	P-value	Estimate	S.E.	Z	P-value	Estimate	S.E.	Z	P-value
Intercept	-0.112	0.174	-0.644	n.s.	0.620	0.237	2.616	0.009	0.197	0.274	0.72	n.s.
Gender	-0.012	0.052	-0.234	n.s.	-0.191	0.071	-2.707	0.007	-0.166	0.082	-2.03	n.s.
Age	0.008	0.002	4.947	<0.001	-0.004	0.002	-1.700	n.s.	0.005	0.002	2.117	n.s.
Years of schooling (<8 years)	-0.005	0.085	-0.055	n.s.	-0.243	0.115	-2.105	n.s.	0.023	0.134	0.171	n.s.
Years of schooling (12 years)	0.221	0.072	3.075	0.002	0.345	0.098	3.532	<0.001	0.033	0.113	0.289	n.s.
Years of schooling (technical complete / higher incomplete)	0.166	0.076	2.188	n.s.	0.446	0.104	4.301	<0.001	-0.047	0.12	-0.396	n.s.
Years of schooling (higher complete)	0.390	0.096	4.059	<0.001	0.346	0.128	2.697	0.007	0.316	0.148	2.137	n.s.
Number of diseases	0.067	0.022	3.046	0.002	0.110	0.030	3.706	<0.001	0.044	0.034	1.304	n.s.
Health status (bad)	-0.205	0.097	-2.112	n.s.	-0.033	0.134	-0.248	n.s.	-0.166	0.155	-1.069	n.s.
Health status (good)	0.038	0.056	0.670	n.s.	0.033	0.077	0.435	n.s.	-0.063	0.089	-0.708	n.s.
Health status (very good)	0.510	0.099	5.129	<0.001	0.428	0.130	3.284	0.001	0.569	0.148	3.85	<0.001
% Multidimensional Poverty Index by commune	0.374	0.439	0.851	n.s.	-0.789	0.595	-1.326	n.s.	0.712	0.687	1.037	n.s.
Phi	6.693	0.297	22.51	<0.001	2.671	0.111	24.05	<0.001	1.438	0.057	25.15	<0.001
Pseudo R-squared	0.081				0.069				0.035			
	General Self-Management and Daily Routines				Drug Access and Intake				Monitoring and Decision-Making			
	Estimate	S.E.	Z	P-value	Estimate	S.E.	Z	P-value	Estimate	S.E.	Z	P-value
Intercept	-0.702	0.21	-3.338	0.001	1.133	0.263	4.315	<0.001	-1.070	0.293	-3.646	<0.001
Gender	0.007	0.063	0.114	n.s.	0.006	0.077	0.074	n.s.	0.047	0.087	0.538	n.s.
Age	0.019	0.002	9.918	<0.001	0.005	0.002	2.039	n.s.	0.010	0.003	3.768	<0.001
Years of schooling (<8 years)	0.058	0.103	0.557	n.s.	0.021	0.127	0.164	n.s.	-0.030	0.144	-0.208	n.s.
Years of schooling (12 years)	0.220	0.087	2.535	n.s.	0.152	0.107	1.417	n.s.	0.251	0.121	2.072	n.s.
Years of schooling (technical complete / higher incomplete)	0.095	0.092	1.039	n.s.	0.058	0.114	0.505	n.s.	0.184	0.128	1.436	n.s.
Years of schooling (higher complete)	0.315	0.115	2.749	0.006	0.191	0.140	1.361	n.s.	0.417	0.158	2.633	n.s.
Number of diseases	-0.030	0.026	-1.142	n.s.	0.064	0.032	1.973	n.s.	0.100	0.037	2.730	0.006
Health status (bad)	-0.348	0.116	-2.991	0.003	-0.221	0.148	-1.492	n.s.	-0.126	0.166	-0.755	n.s.
Health status (good)	0.255	0.068	3.749	<0.001	0.064	0.084	0.762	n.s.	0.042	0.095	0.440	n.s.
Health status (very good)	0.756	0.119	6.368	<0.001	0.224	0.141	1.586	n.s.	0.417	0.159	2.630	n.s.
% Multidimensional Poverty Index by commune	0.425	0.528	0.806	n.s.	-0.020	0.653	-0.031	n.s.	1.520	0.736	2.063	n.s.
Phi	4.026	0.175	23.010	<0.001	1.428	0.076	18.76	<0.001	0.955	0.036	26.620	<0.001
Pseudo R-squared	0.130				0.035				0.042			

S.E.: standard error.

Table 4 presents the results of sequential ANOVA using the residual values obtained from the beta regressions, with the aim of controlling for the other independent variables. The association between SMM and the independent variables remained similar, while DK was significantly associated with years of schooling and number of diseases, SMDR was significantly associated with age and health status, DAI was significantly associated with health status, and MDM was significantly associated with age. HTR showed no association with the independent variables. Eta squared showed small effect sizes of each independent variable on SMM and its dimensions.

**Table 4:** Sequential ANOVA of self-management mean and its dimensions with the independent variables.

Variable	Self-Management Mean			Disease Knowledge			Healthcare Team Relationship		
	F value	P-value	Eta-squared	F value	P-value	Eta-squared	F value	P-value	Eta-squared
Gender	0.036	n.s.	<0.001	5.099	n.s.	0.006	3.148	n.s.	0.003
Age	15.790	<0.001	0.017	2.055	n.s.	0.002	2.689	n.s.	0.003
Years of schooling	11.040	0.001	0.012	17.070	<0.001	0.018	0.905	n.s.	0.001
Number of diseases	7.607	0.006	0.008	8.672	0.003	0.009	1.169	n.s.	0.001
Health status	19.960	<0.001	0.022	4.222	n.s.	0.005	5.900	n.s.	0.006
% Multidimensional Poverty Index by commune	0.154	n.s.	<0.001	1.765	n.s.	0.002	0.371	n.s.	<0.001
	General Self-Management and Daily Routines			Drug Access and Intake			Monitoring and Decision-Making		
	F value	P-value	Eta-squared	F value	P-value	Eta-squared	F value	P-value	Eta-squared
Gender	0.011	n.s.	<0.001	0.010	n.s.	<0.001	0.247	n.s.	<0.001
Age	52.930	<0.001	0.055	5.511	n.s.	0.006	9.934	0.002	0.011
Years of schooling	2.588	n.s.	0.003	1.805	n.s.	0.002	4.877	n.s.	0.005
Number of diseases	0.662	n.s.	0.001	6.240	n.s.	0.007	5.848	n.s.	0.006
Health status	40.580	<0.001	0.043	8.693	0.003	0.010	4.583	n.s.	0.005
% Multidimensional Poverty Index by commune	0.041	n.s.	<0.001	0.002	n.s.	<0.001	2.925	n.s.	0.003

## Discussion

Self-management is determined by multiple variables at various levels [20–22] and this study examined how of variation in sociodemographic and health factors impacted on self-management.

This study used a modified PIH scale to monitor how participants, during the Covid-19 pandemic, expressed their abilities to perform tasks by themselves and what they received from their support networks and healthcare systems. Although the SMM was observed to have a high score, its dimensions showed heterogeneity. For example, the DAI dimension showed that actions conducted with institutional support mechanisms established prior to the Covid-19 pandemic, presented higher scores and low variability. On the contrary, SMDR which includes physical activity and diet behaviors that depend on people and their environment presented lower scores and were more variable. These results can be explained by the difficulty in performing physical activity and diet-related behaviors compared to medication intake.

The SMM model accounted for 8.1% of the variation of the self-management construct, while the independent variables explained between 3.5% to 13% of the studied dimensions indicating that there is an important amount of unexplained variation. Other studies [35–37] found that the nature of social support, self-efficacy, cultural values, self-awareness, empowerment, perceived barriers, health literacy, among others, improved self-management, thereby accounting for the higher percentage of variation explained in these studies.

In the present study, age, years of schooling level, number of diseases and self-rated health status were significantly associated with SMM, DK, SMDR, and MDM. These variables have been used in other research [38–45] aiming to understand the short-term modifiable effects on self-management.

Self-rated health status was statistically associated with SMM and the SMDR dimension. Additionally, after removing the effect of the other independent variables, self-rated health status presented a significant association with the DAI dimension. These results are consistent with previous studies, which found that self-rated health status is a variable significantly associated with other health indicators. Smith et al. [43] demonstrated that self-rated health was significantly associated with self-management measured with the PIH scale. The authors concluded that the probability of having high self-management is greater if the participant has a good or excellent self-rated health status. The significant association that exists between these two variables can be explained as a positive reciprocal influence, where better disease self-management generates improved self-rated health status, reinforcing self-management behaviors.

Years of schooling showed a significant association with SMM, as well as with the DK, SMDR, HTR, and MDM dimensions with the higher education category showing the largest contribution to higher self-management. This could be explained by a greater capacity of the population to understand messages, information, and in-depth reflection regarding prescriptions provided by healthcare teams. During the Covid-19 pandemic, this skill appears as an accumulated history that should be taken into account by healthcare teams when providing care to users. Heijmans et al. [38] found in a Dutch study that education level significantly influences healthcare literacy and consequently, self-management. Heijmans et al. [38] argued that communication, functional and critical thinking skills in the population that access healthcare services are a relevant resource for the structural foundations of a healthcare system. Kim et al. [46] proposed that it is important to consider the broad scope and skill-depth that participants can develop for self-management, the paths to search and obtain information, and how health information can be applied in their daily lives, which requires knowledge, motivational skills, access to official information and the capacity to develop strategies to implement sustainable changes.

Age showed significant and positive associations with SMM, SMDR and MDM. This suggests that the accumulation of advice and experience, routines and resilience acquired from the process of living with a NCD leads to improvements in disease management, which are expressed in concrete actions taken during Covid-19 lockdowns, including diet, physical activity and emotional management. The significant association between age and the MDM dimension reveals that age is positively correlated with greater performance in monitoring signs and symptoms and making decisions based on these assessments. These results are similar to those reported by Heijmans et al. [38], who also using the PIH scale, observed a positive association between age and self-management.

The number of diseases presented a positive association with SMM and the DK dimensions. Participants with a higher number of diseases have greater self-management and disease knowledge. Other studies [13, 38, 47] have observed that the number of diseases has a significant inverse effect on self-management. Fix et al. [47] observed that patients with comorbidities perceive an interdependence between their conditions and subsequently had problems separating information between their illnesses. However, the majority of conditions shared by participants in this study have a common set of characteristics (origins, indications, consequences and manner to be addressed by the healthcare system). Consequently, information and indications received for one disease can function as reinforcement for others leading to greater levels of knowledge and self-management. The results obtained from this study and previous literature [13, 38, 47] suggest that future research should consider the total number of diseases, as well as groups of diseases, in order to know the differential effect on self-management and its dimensions.

In this study self-reported gender was not significantly associated with SMM or its dimensions, which suggests that self-perception of practices, knowledge and experiences learned from living with a condition are not mediated by gender perception [48]. The effect of gender on NCD self-management is not consistent and some studies have observed significant associations [39, 41, 49, 50] while others have not [43, 51, 52], or an association that is exclusive to specific dimensions [38]. An explanation for these disparities in findings could be attributed to different cultural expectations associated with gender roles in the countries where studies were conducted. For instance, Alrahbi [49] observed that Omani men performed physical activity more regularly than women, due to social restrictions imposed on women. Hara et al. [50] observed a worsening of psychological symptoms associated with diabetes in women, which suggests that gender has a major impact on patient empowerment and that female patients may face more psychological or family communication issues. Both studies aim at social and cultural differences that are involved in NCD self-management. The different ways that men and women address their own health has been acknowledged and studied [43] and WHO [53] acknowledges the effect of gender on general health. Health practices and behaviors can be understood as activities built in association with gender roles [54, 55]. One possible explanation of the present result is that the PSCV program provides homogenous healthcare to men and women alike, without differentiating care in accordance to gender characteristics, and as a possible result, the response that is received is also homogenous.

There was no association between self-management and the extent of commune poverty in the present study. This lack of association could be due to the low variation in % poverty between communes and that household income might have been a better predictor. For example, the National Socioeconomic Characterization Survey (Encuesta de Caracterización Socioeconómica Nacional) 2017 [56] found a near two-fold increase in poverty and extreme poverty levels by household income, in the Región Metropolitana [57].

Finally, the HTR dimension did not show significant association with any independent variables. There is evidence that indicates that the relationship between healthcare teams and participants are determined by variables such as personality traits, communication skills and certain common characteristics [58, 59], which were not assessed in our study.

Our study has several limitations. Data acquired through a telephone survey, may affect the results compared to face-to-face survey. However, the limitations imposed by the lock-down due to the Covid-19 pandemic, prevented any type of in-person meetings. Another limitation is the cross-sectional nature of this research. The research was carried out between the first and the second waves of Covid-19 in Chile and it is not known whether self-management changed during this time due to the changing access to healthcare or support strategies. Additionally, this research did not consider other types of independent variables, such as other social and individual variables, cultural variables, or psychological conditions, among others. However, the purpose of this research was to focus understanding on the effects of selected sociodemographic and health factors in order to know what kind of social and individual barriers or enhancers have to be considered to implement strategies to improve the self-management. Finally, it is possible that some bias may have occurred due to refusal of some participants to take part in the research. While gender, age, and commune of residence were controlled by the sample design, other variables such as SMM and its dimensions, as well as years of schooling, number of diseases, and health status were not controlled, showing eventual differences between the participants and those who refused to participate.

## Conclusions

Self-management is a complex phenomenon determined by a large number of variables of diverse nature, which interact with each other.

The Covid-19 pandemic interrupted the regular health care system and this situation provided an opportunity to study the role that the health systems has in relationship to self-management in the context of NCDs.

This study showed that when the health system develops and consolidate practices focused on self-management, such as drug access, there is stability. On the contrary, other individual dependent dimensions that have not been reinforced before the pandemic, showed more differential responses and the PSCV needs to recognize its strengths and weaknesses in order to improve self-management as a crucial factor in the Chronic Care Model [57] adopted by the PSCV.

At the global level, this study shows some directions to improve the quality of healthcare systems [58] in countries experiencing crises. Strategies linked to self-management support should focus on providing structural and community mechanisms that allow strengthening self-management in the population before, during and after a pandemic or in future health, social or natural disaster crises, taking into consideration sociodemographic and health factors that modulate the expression of self-management.

## Supporting information

S1 Text Questionnaire. Chronic conditions self-management during Covid-19 pandemic.
(DOCX)

## References

[1] PAHO Noncommunicable diseases. Regional office for the Americas WHO [Internet]. https://www.paho.org/es/temas/enfermedades-no-transmisibles.

[2] Yang C, Jin Z. An Acute Respiratory Infection runs into the most common noncommunicable Epidemic- COVID-19 and Cardiovascular diseases. JAMA Cardiol 2020; 5(7):743–744. doi: 10.1001/jamacardio.2020.0934 32211809

[3] World Health Organization. Innovative Care for Chronic Conditions: Building Blocks for Action [Internet]. 2002. https://apps.who.int/iris/handle/10665/42500.

[4] World Health Organization Noncommunicable diseases [Internet]. 2021. https://www.who.int/news-room/fact-sheets/detail/noncommunicable-diseases#:~:text=Noncommunicable%20diseases%20(NCDs)%2C%20also,physiological%2C%20environmental%20and%20behavioural%20factors.

[5] Clark A, Jit M, Warren- Gash C, Guthrie B, Wang HH, Mercer SW, et al. Global, regional and national estimates of the population at increased risk of severe COVID- 19 due to underlying conditions in 2020: a modelling study. Lancet Glob Health 2020 Aug; 8(8):e1003–e1017. doi: 10.1016/S2214-109X(20)30264-3 32553130 PMC7295519

[6] Clerkin K, Fried J, Raikhelkar J, Sayer G, Griffin J, Masoumi A, et al. COVID- 19 and Cardiovascular Disease. Circulation 2020 May 19; (141)20:1648–1655. doi: 10.1161/CIRCULATIONAHA.120.046941 32200663

[7] Bakouny Z, Hawley JE, Choueiri TK, Peters S, Rini BI, Warner JL, et al. COVID-19 and Cancer: Current Challenges and Perspectives. Cancer Cell. 2020 Nov 9;38(5):629–646. Epub 2020 Oct 1. doi: 10.1016/j.ccell.2020.09.018 33049215 PMC7528740

[8] Kluge HHP, Wickramasinghe K, Rippin HL, Mendes R, Peters DH, Kontsevaya A, et al. Prevention and control of non-communicable diseases in the COVID- 19 response. The Lancet 2020 May 30;395(10238):1678–1680. doi: 10.1016/S0140-6736(20)31067-9 32401713 PMC7211494

[9] Medical College of Chile, Universidad Diego Portales, Pontificia Universidad Católica de Chile, Universidad San Sebastián, Universidad Central, Universidad de la Frontera. MCOVID-19 ¿Cuál ha sido el impacto de la pandemia en el acceso a atenciones de salud? Un análisis para la adaptación de nuestro sistema de salud. Santiago, Chile. [Internet] 2020 Oct 5. https://www.movid19.cl/publicaciones/decimo-informe/.

[10] Latorre R. Healthcare resorts to Primary Care after hospital collapse and enables “prolonged observation” beds in clinics [Internet] La Tercera. 2021 Jun 9. https://www.latercera.com/earlyaccess/noticia/salud-recurre-a-la-atencion-primaria-tras-colapso-hospitalario-y-habilita-camas-de-observacion-prolongada-en-consultorios/KQ2FSNM2P5CIBJH25MKE4RLEFE/.

[11] Ministerio de Salud, Gobierno de Chile. Orientación Técnica Programa de Salud Cardiovascular. [Internet] 2017. http://familiarycomunitaria.cl/FyC/wp-content/uploads/2018/05/Programa-de-salud-cardiovascular.-MINSAL-Chile-2017.pdf.

[12] Kastner M, Hayden L, Wong G, Lai Y, Makarski J, Treister V, et al. Underlying mechanisms of complex interventions addressing the care of older adults with multimorbidity: a realist review. BMJ Open. 2019 Apr 3;9(4):e025009. doi: 10.1136/bmjopen-2018-025009 30948577 PMC6500199

[13] May CR, Eton DT, Boehmer K, Gallacher K, Hunt K, MacDonald S, et al. Rethinking the patient: using Burden of Treatment Theory to understand the changing dynamics of illness. Bmc Health Serv Res. 2014 Jun 26;14(1):281. doi: 10.1186/1472-6963-14-281 24969758 PMC4080515

[14] Hardman R, Begg S, Spelten E. What impact do chronic disease self-management support interventions have on health inequity gaps related to socioeconomic status: a systematic review. BMC Health Serv Res 2020 Feb 27;20(1):150. doi: 10.1186/s12913-020-5010-4 32106889 PMC7045733

[15] Corbin J, Strauss A. Managing Chronic Illness at home: three lines of work. Qual Sociol. 1985 Sep;8:224–247.

[16] Coleman MT, Newton KS. Supporting self-management in patients with chronic illness. Am Fam Physician. 2005 Oct 15;72(8):1503–10. . 16273817

[17] Pearce G, Parke HL, Pinnock H, Epiphaniou E, Bourne CL, Sheikh A, et al. The PRISMS taxonomy of self-management support: derivation of a novel taxonomy and initial testing of its utility. J Health Serv Res Policy. 2016 Apr;21(2):73–82. Epub 2015 Sep 15. doi: 10.1177/1355819615602725 . 26377727

[18] World Health Organization. Preparation of Health Care Professionals for the 21st Century. The Challenge of Chronic Diseases, World Health Organization. 2005. https://apps.who.int/iris/handle/10665/43044

[19] Bonal RR, López VN, Vargas P, Meoño MT, Brañas CRW. Support to Chronic Conditions Self-management: a Challenge of Health Systems in Latin America. Finlay. 2017;7(4):268–277. Available from https://www.medigraphic.com/cgi-bin/new/resumenI.cgi?IDARTICULO=77124.

[20] Ochieng JM, Crist JD. Social Determinants of Health and Health Care Delivery: African American Women’s T2DM Self-Management. Clin Nurs Res. 2021 Mar;30(3):263–272. Epub 2020 Apr 22. doi: 10.1177/1054773820916981 . 32321292

[21] Hyman I, Shakya Y, Jembere N, Gucciardi E, Vissandjée B. Provider- and patient-related determinants of diabetes self-management among recent immigrants: Implications for systemic change. Can Fam Physician. 2017 Feb;63(2):e137–e144. 28209706 PMC5395412

[22] Khalesi S, Irwin C, Sun J. Lifestyle and self-management determinants of hypertension control in a sample of Australian adults. Expert Rev Cardiovasc Ther. 2018 Mar;16(3):229–236. Epub 2018 Feb 5. doi: 10.1080/14779072.2018.1435272 . 29388449

[23] OECD. OECD Reviews of Public Health: Chile: A Healthier Tomorrow. 2019. OECD Publishing, Paris.

[24] Potoglou D, Kanaroglou PS, Robinson N. Evidence on the Comparison of Telephone and Internet Surveys for Respondent Recruitment. The Open Transportation Journal. 2012;6(1):11–22.

[25] Manfreda KL, Bosnjak M, Berzelak J, Haas I, Vehovar V. Web Surveys versus other Survey Modes: A Meta-Analysis Comparing Response Rates. International Journal of Market Research. 2008;50(1):79–104. doi: 10.1177/147078530805000107

[26] Battersby W, Ask A, Reece M, Markwick J, Collins P. The Partners in Health scale: The development and psychometric properties of a generic assessment scale for chronic condition self-management. Australian Journal of Primary Health. 2003;9(3):41–52. Available from: https://www.publish.csiro.au/PY/ExportCitation/PY03022

[27] Bandura A. Guide for constructing self-efficacy scales. In: Pajares F, Urdan T, editors. Self-efficacy beliefs of adolescents. 5. Greenwich, CT: Information Age Publishing; 2006. p. 307–37.

[28] Breland JY, Wong JJ, McAndrew LM. Are Common Sense Model constructs and self-efficacy simultaneously correlated with self-management behaviors and health outcomes: A systematic review. Health Psychol Open. 2020;7(1):2055102919898846-. doi: 10.1177/2055102919898846 32030192 PMC6978827

[29] Banerjee A, Hendrick P, Bhattacharjee P, Blake H. A systematic review of outcome measures utilised to assess self-management in clinical trials in patients with chronic pain. Patient Educ Couns. 2018 May;101(5):767–778. Epub 2017 Dec 10. doi: 10.1016/j.pec.2017.12.002 . 29258726

[30] Willis GB. Analysis of the cognitive interview in questionnaire design. Oxford University Press. 2015.

[31] Cribari-Neto F, Zeileis A. Beta Regression in R. Journal of Statistical Software. 2010;34(2):1–24.

[32] Noel Y, Dauvier B. A Beta Item Response Model for Continuous Bounded Responses. Applied Psychological Measurement. 2007;31(1):47–73

[33] Verkuilen J, Smithson M. Mixed and Mixture Regression Models for Continuous Bounded Responses Using the Beta Distribution. Journal of Educational and Behavioral Statistics. 2012;37(1):82–113.

[34] Niekerk JV, Bekker A, Arashi M. Beta regression in the presence of outliers—A wieldy Bayesian solution. Stat Methods Med Res. 2019 Dec;28(12):3729–3740. Epub 2018 Nov 25. doi: 10.1177/0962280218814574 . 30474473

[35] Duman JGY. Self-Management of Chronic Diseases: A Descriptive Phenomenological Study. Soc Work Public Health. 2021 Feb 17;36(2):300–310. Epub 2020 Dec 30. doi: 10.1080/19371918.2020.1859034 . 33378254

[36] Thojampa S, Mawn B. The moderating effect of social cognitive factors on self-management activities and HbA1c in Thai adults with type-2 diabetes. Int J Nurs Sci. 2016 Dec 30;4(1):34–37. doi: 10.1016/j.ijnss.2016.12.006 31406715 PMC6626080

[37] Yang L, Li K, Liang Y, Zhao Q, Cui D, Zhu X. Mediating role diet self-efficacy plays in the relationship between social support and diet self-management for patients with type 2 diabetes. Arch Public Health. 2021 Jan 31;79(1):14. doi: 10.1186/s13690-021-00533-3 33517902 PMC7849071

[38] Heijmans M, Waverijn G, Rademakers J, van der Vaart R, Rijken M. Functional, communicative and critical health literacy of chronic disease patients and their importance for self-management. Patient Educ Couns. 2015 Jan;98(1):41–8. Epub 2014 Oct 16. doi: 10.1016/j.pec.2014.10.006 . 25455794

[39] Albright TL, Parchman M, Burge SK; RRNeST Investigators. Predictors of self-care behavior in adults with type 2 diabetes: an RRNeST study. Fam Med. 2001 May;33(5):354–60. . 11355645

[40] Battersby M, Harris M, Smith D, Reed R, Woodman R. A pragmatic randomized controlled trial of the Flinders Program of chronic condition management in community health care services. Patient Educ Couns. 2015 Nov;98(11):1367–75. Epub 2015 Jun 17. doi: 10.1016/j.pec.2015.06.003 . 26146240

[41] Lee LY, Tung HH, Tsay SL, Chen YC, Lee HH, Zeng YX. Predictors for self-management in older adults with type 2 diabetic nephropathy. J Clin Nurs. 2020 Mar;29(5–6):922–931. Epub 2020 Jan 10. doi: 10.1111/jocn.15154 . 31876037

[42] Smith D, Lawn S, Harvey P, Battersby M. Concurrent validity of the Partners in Health scale against general self-rated health in chronic conditions: A short report. Chronic Illn. 2019 Mar;15(1):74–77. Epub 2017 Nov 24. doi: 10.1177/1742395317743559 . 29171291

[43] Smith D, Fairweather-Schmidt AK, Harvey P, Bowden J, Lawn S, Battersby M. Does the Partners in Health scale allow meaningful comparisons of chronic condition self-management between men and women? Testing measurement invariance. J Adv Nurs. 2019 Nov;75(11):3126–3137. Epub 2019 Jul 21. doi: 10.1111/jan.14124 . 31236969

[44] Schüz N, Walters JA, Cameron-Tucker H, Scott J, Wood-Baker R, Walters EH. Patient Anxiety and Depression Moderate the Effects of Increased Self-management Knowledge on Physical Activity: A Secondary Analysis of a Randomised Controlled Trial on Health-Mentoring in COPD. COPD. 2015;12(5):502–9. Epub 2015 Mar 16. doi: 10.3109/15412555.2014.995289 . 25774660

[45] Vaughan B, Grant M, Moroz J, Ngawaka C, Mulcahy J. Self-management behaviour and knowledge of patients with musculoskeletal complaints attending an Australian osteopathy clinic: A consecutive sampling design. International Journal of Osteopathic Medicine. 2020 37:3–9. doi: 10.1016/j.ijosm.2020.05.004

[46] Kim S, Song Y, Park J, Utz S. Patients’ Experiences of Diabetes Self-Management Education According to Health-Literacy Levels. Clin Nurs Res. 2020 Jun;29(5):285–292. Epub 2019 Aug 9. doi: 10.1177/1054773819865879 . 31394916

[47] Fix GM, Cohn ES, Solomon JL, Cortés DE, Mueller N, Kressin NR, et al. The role of comorbidities in patients’ hypertension self-management. Chronic Illn. 2014 Jun;10(2):81–92. Epub 2013 Jul 26. doi: 10.1177/1742395313496591 23892774 PMC8887829

[48] Robertson S. Understanding men and health: masculinities, identity, and well-being. Maidenhead: Open University Press. 2007.

[49] Alrahbi H. Diabetes self-management (DSM) in Omani with type-2 diabetes. International Journal of Nursing Sciences. 2014;1(4):352–9.

[50] Hara Y, Iwashita S, Okada A, Tajiri Y, Nakayama H, Kato T, et al. Development of a novel, short, self-completed questionnaire on empowerment for patients with type 2 diabetes mellitus and an analysis of factors affecting patient empowerment. Biopsychosoc Med. 2014 Aug 25;8:19. doi: 10.1186/1751-0759-8-19 25183994 PMC4151376

[51] Kurnia AD, Amatayakul A, Karuncharernpanit S. Predictors of diabetes self-management among type 2 diabetics in Indonesia: Application theory of the health promotion model. Int J Nurs Sci. 2017 Jul 6;4(3):260–265. doi: 10.1016/j.ijnss.2017.06.010 31406750 PMC6626170

[52] D’Souza MS, Karkada SN, Parahoo K, Venkatesaperumal R, Achora S, Cayaban ARR. Self-efficacy and self-care behaviours among adults with type 2 diabetes. Appl Nurs Res. 2017 Aug;36:25–32. Epub 2017 May 22. doi: 10.1016/j.apnr.2017.05.004 . 28720235

[53] World Health Organization. Gender and Health. [Consulted 2021 jul 15]. https://www.who.int/health-topics/gender#tab=tab_1.

[54] Saltonstall R. Healthy bodies, social bodies: men’s and women’s concepts and practices of health in everyday life. Soc Sci Med. 1993 Jan;36(1):7–14. doi: 10.1016/0277-9536(93)90300-s . 8424186

[55] Robertson S. Understanding men and health: Masculinities, identity and well-being: Masculinity, identity and well-being. McGraw-Hill Education. UK. 2007.

[56] Ministry of Social Development, Government of Chile. Sample design methodology. 2018. http://observatorio.ministeriodesarrollosocial.gob.cl/casen-multidimensional/casen/docs/Diseno_Muestral_Casen_2017_MDS.pdf

[57] Ministry of Social Development 2020. Summary of results: Poverty by income and income distribution. CASEN 2020. Social Observatory. Santiago, Chile. http://observatorio.ministeriodesarrollosocial.gob.cl.

[58] Chandra S, Mohammadnezhad M, Ward P. Trust and Communication in a Doctor- Patient Relationship: A Literature Review. Journal of Healthcare Communications. 2018. 3:36, doi: 10.4172/2472-1654.100146

[59] Chipidza FE, Wallwork RS, Stern TA. Impact of the Doctor-Patient Relationship. Prim Care Companion CNS Disord. 2015 Oct 22;17(5). doi: 10.4088/PCC.15f01840 26835164 PMC4732308

