# Correction: Effects of sociodemographic and health factors on the self-management of non-communicable diseases among Chilean adults during the Covid-19 pandemic

**DOI:** 10.1371/journal.pgph.0002512

**Published:** 2023-10-11

**Authors:** Daniela Nicoletti-Rojas, Rodrigo Retamal, Ricardo Cerda-Rioseco, Lorena Rodríguez-Osiac, Mauricio Fuentes-Alburquenque, Marcela Araya-Bannout

There is an error in the current address for author Ricardo Cerda-Rioseco. The correct current address is: Facultad de Medicina, Pontificia Universidad Católica de Chile, Santiago, Región Metropolitana, Chile. There is also an error in affiliation 4 for author Ricardo Cerda-Rioseco. The correct affiliation 4 is: Departamento de Ciencias de la Salud, Facultad de Medicina, Pontificia Universidad Católica de Chile, Santiago, Región Metropolitana, Chile.
