# Supplementary material for: Effects of sociodemographic and health factors on the self-management of non-communicable diseases among Chilean adults during the Covid-19 pandemic
Source: PLOS Glob Public Health. 2022 Jul 21;2(7):e0000763. doi: 10.1371/journal.pgph.0000763 (PMC10021505; doi:10.1371/journal.pgph.0000763)
Supplement: S1 Text — Chronic conditions self-management during Covid-19 pandemic. (DOCX) [file pgph.0000763.s001.docx]

**QUESTIONNAIRE**

**CHRONIC CONDITIONS SELF-MANAGEMENT DURING COVID-19 PANDEMIC.**

**Project: ANID COVID0139**

Instructions:

Due to the pandemic, we are interested in how you have managed your chronic conditions in this context. Below there are a series of statements that refer to different aspects of managing your condition(s) or chronic disease (s)(diabetes and/or hypertension) since we are in a pandemic.

For each statement I am going to ask you to indicate how much you handle these aspects from 0 to 100%, where 0 means that you cannot handle it al all and 100, that you handle it perfectly, being able to score any number between them (Explain that 50 means intermediate driving, which 10 or 20 means driving a little, etc).

Please respond as calmly and honestly as possible, there are no good or bad answers, remember that you are not being evaluated.

***Thinking in your chronic conditions, such as Diabetes and Hypertension, from 0 to 100 ...***

| Question | Absence of self-management Complete self-management |
| --- | --- |
| 1. ¿How much you know about your disease (s)? | 0        10       20         30         40        50          60             70          80         90        100 |
| 2. ¿How much you know about the causes of your disease (s)? | 0        10       20         30         40        50          60             70          80         90        100 |
| 3. ¿How much you know about the treatment of your disease (s)? | 0        10       20         30         40        50          60             70          80         90        100 |
| 4. ¿How much you know about the effect of your disease (s) in coronavirus? | 0        10       20         30         40        50          60             70          80         90        100 |
| 5. ¿To what extent does the health team consider your opinion when giving your treatment? | 0        10       20         30         40        50          60             70          80         90        100 |
| 6. ¿How important is your opinion in your treatment? | 0        10       20         30         40        50          60             70          80         90        100 |
| 7. ¿To what extent the health team supports you in the pandemic? | 0        10       20         30         40        50          60             70          80         90        100 |
| 8 ¿To what extent can you assist to your health appointments at the cesfam in the pandemic? | 0        10       20         30         40        50          60             70          80         90        100 |
| 9. ¿To what extent are you able to access your medications during the pandemic? | 0        10       20         30         40        50          60             70          80         90        100 |
| 10. In pandemic ¿To what extent are you able to take your medications for chronic conditions, as prescribed by the doctor/ health team? | 0        10       20         30         40        50          60             70          80         90        100 |
| 11. ¿To what extent can you monitor your BP and/or Glycemia, as indicated by the health team? | 0        10       20         30         40        50          60           70          80         90        100 |
| 12. ¿To what extent do you know what to do when the signs and symptoms of your disease (s) get worse? | 0        10       20         30         40        50          60           70          80         90        100 |
| 13. ¿How much do you handle the daily self-care of your disease (s) in the pandemic? | 0        10       20         30         40        50          60           70          80         90        100 |
| 14. ¿How much you handle your diet as indicated by the health team in pandemic? | 0        10       20         30         40        50          60           70          80         90        100 |
| 15. ¿How much you handle your physical activity as indicated by the health team in pandemic? | 0        10       20         30         40        50          60           70          80         90        100 |
| 16. ¿How much you can count on your family, friends or neighbors when you need help? | 0        10       20         30         40        50          60           70          80         90        100 |
| 17. ¿How much you manage your emotions living with this disease (s) in pandemic? | 0        10       20         30         40        50          60           70          80         90        100 |

Dimensions obtained from factor analysis and contained items

| **Dimension** | **Item** | **Question** |
| --- | --- | --- |
| Disease Knowledge | A1 | ¿How much you know about your disease (s)? |
|  | A2 | ¿How much you know about the causes of your disease (s)? |
|  | A3 | ¿How much you know about the treatment of your disease (s)? |
|  | A4 | ¿How much you know about the effect of your disease (s) in coronavirus? |
| Healthcare Team Relationship | A5 | ¿To what extent does the health team consider your opinion when giving your treatment? |
|  | A6 | ¿How important is your opinion in your treatment? |
|  | A7 | ¿To what extent the health team supports you in the pandemic? |
| General Self-Management and Daily Routines | A13 | ¿How much do you handle the daily self-care of your disease (s) in the pandemic? |
|  | A14 | ¿How much you handle your diet as indicated by the health team in pandemic? |
|  | A15 | ¿How much you handle your physical activity as indicated by the health team in pandemic? |
|  | A17 | ¿How much you manage your emotions living with this disease (s) in pandemic? |
| Drug Access and Intake | A9 | ¿To what extent are you able to access your medications during the pandemic? |
|  | A10 | In pandemic ¿To what extent are you able to take your medications for chronic conditions, as prescribed by the doctor/ health team? |
| Monitoring and Decision-Making | A11 | ¿To what extent can you monitor your BP and/or Glycemia, as indicated by the health team? |
|  | A12 | ¿To what extent do you know what to do when the signs and symptoms of your disease (s) get worse? |

**CUESTIONARIO**

**AUTOMANEJO DE CONDICIONES CRÓNICAS DURANTE LA PANDEMIA POR COVID- 19**

**Proyecto ANID COVID0139**

**Instrucciones:**

Dado que estamos en pandemia, nos interesa saber cómo ha podido manejar sus condiciones crónicas en este contexto. A continuación, se presentan una serie de afirmaciones que se refieren a diferentes aspectos del manejo de su (s) condición (es) o enfermedad (es) crónica (s), como la diabetes y la Hipertensión*,* desde que estamos en pandemia.

Le voy a pedir que, frente a cada afirmación, indique cuanto logra manejar estos aspectos de 0 a 100%, donde 0 significa que no puede manejarlo y 100, que lo maneja perfectamente, pudiendo también puntuar cualquier cifra entre ambas (Explicar que 50 significa un manejo intermedio, que 10 o 20 significa que maneja un poco, etc).

Le pido por favor que responda de la forma más tranquila y honesta posible, no existen respuestas buenas ni malas, recuerde que no está siendo evaluado.

*Pensando sólo en sus condiciones crónicas, como Diabetes e Hipertensión, de 0 a 100…*

| Pregunta | Ausencia de automanejo Automanejo completo |
| --- | --- |
| 1. ¿Cuánto sabe sobre su (s) enfermedad (es)? | 0        10       20         30         40        50          60           70          80         90        100 |
| 2. Cuánto sabe sobre las causas de su (s) enfermedad (es)? | 0        10       20         30         40        50          60           70          80         90        100 |
| 3. ¿Cuánto sabe sobre el tratamiento de sus (s) enfermedad (es)? | 0        10       20         30         40        50          60           70          80         90        100 |
| 4. ¿Cuánto sabe sobre el efecto de su (s) enfermedad (es) en el coronavirus? | 0        10       20         30         40        50          60           70          80         90        100 |
| 5. En qué medida el equipo de salud considera su opinión al darle el tratamiento? | 0        10       20         30         40        50          60           70          80         90        100 |
| 6. Qué importancia tiene su opinión dentro de su tratamiento? | 0        10       20         30         40        50          60           70          80         90        100 |
| 7. ¿En qué medida el equipo médico es un apoyo para usted en la pandemia? | 0        10       20         30         40        50          60           70          80         90        100 |
| 8 ¿En qué medida tiene la posibilidad de acudir a controles al cesfam durante la pandemia? | 0        10       20         30         40        50          60           70          80         90        100 |
| 9. En qué medida tiene la posibilidad de acceder a los medicamentos que necesita para su (s) enfermedad (es) durante la pandemia? | 0        10       20         30         40        50          60           70          80         90        100 |
| 10. En pandemia, en qué medida logra tomarse sus medicamentos para las condiciones crónicas, tal como lo tiene indicado por el médico/ equipo médico? | 0        10       20         30         40        50          60           70          80         90        100 |
| 11. ¿En qué medida logra hacer seguimiento a su PA y/o Glicemia, tal como le indicaron en el cesfam? | 0        10       20         30         40        50          60           70          80         90        100 |

| 12. En qué medida sabe qué hacer cuando los signos y síntomas de su (s) enfermedad (es) empeoran? | 0        10       20         30         40        50          60           70          80         90        100 |
| --- | --- |
| 13. ¿Cuánto maneja el autocuidado diario de sus enfermedades en la pandemia? | 0        10       20         30         40        50          60           70          80         90        100 |
| 14. ¿Cuánto maneja su alimentación según las indicaciones del equipo médico, en pandemia? | 0        10       20         30         40        50          60           70          80         90        100 |
| 15. ¿Cuánto maneja su actividad física según las indicaciones del equipo médico, en pandemia? | 0        10       20         30         40        50          60           70          80         90        100 |
| 16. ¿En qué medida puede acudir a familiares, amigos o vecinos, cuando necesita ayuda? | 0        10       20         30         40        50          60           70          80         90        100 |
| 17. ¿Cuánto maneja sus emociones al vivir con esta (s) enfermedad (es) en el contexto de pandemia? | 0        10       20         30         40        50          60           70          80         90        100 |

Dimensiones obtenidas del análisis factorial e ítems contenidos

| **Dimensión** | **Ítem** | **Pregunta** |
| --- | --- | --- |
| Conocimiento sobre la(s) enfermedad(es) | A1 | ¿Cuánto sabe sobre su(s) enfermedad(es)? |
|  | A2 | ¿Cuánto sabe sobre las causas de su(s) enfermedad(es)? |
|  | A3 | ¿Cuánto sabe sobre el tratamiento de su(s) enfermedad(es)? |
|  | A4 | ¿Cuánto sabe sobre el efecto de su(s) enfermedad(es) en el coronavirus? |
| Relación con el equipo de salud | A5 | ¿En qué medida el equipo de salud considera su opinión al darle el tratamiento? |
|  | A6 | ¿Qué importancia tiene su opinión dentro de su tratamiento? |
|  | A7 | ¿En qué medida el equipo médico es un apoyo para usted en la pandemia? |
| Automanejo general y de conductas cotidianas | A13 | ¿Cuánto maneja el autocuidado de su(s) enfermedad(es) en pandemia? |
|  | A14 | ¿Cuánto maneja su alimentación según las indicaciones del equipo médico, en pandemia? |
|  | A15 | ¿Cuánto maneja su actividad física según las indicaciones del equipo médico, en pandemia? |
|  | A17 | ¿Cuánto maneja sus emociones al vivir con esta(s) enfermedad(es) en el contexto de la pandemia? |
| Acceso y toma de medicamentos | A9 | ¿En qué medida tiene la posibilidad de acceder a los medicamentos que necesita para su(s) enfermedad(es) durante la pandemia? |
|  | A10 | ¿En qué medida logra tomarse sus medicamentos para su(s) enfermedad(es) crónicas, tal como lo tiene indicado por el médico o equipo médico, durante la pandemia? |
| Monitoreo y saber qué hacer | A11 | ¿En qué medida logra hacer seguimiento a su Presión arterial y/o glicemia tal como le indicaron en el cesfam, en pandemia? |
|  | A12 | ¿En qué medida sabe qué hacer cuando los signos y síntomas de su(s) enfermedad(es) empeoran en pandemia? |
